# Grape Seed Proanthocyanidins play the roles of radioprotection on Normal Lung and radiosensitization on Lung Cancer via differential regulation of the MAPK Signaling Pathway

**DOI:** 10.7150/jca.49987

**Published:** 2021-03-15

**Authors:** Yang Xu, Yijuan Huang, Yuanyuan Chen, Kun Cao, Zhe Liu, Zhijie Wan, Zebin Liao, Bailong Li, Jianguo Cui, Yanyong Yang, Xiaohua Xu, Jianming Cai, Fu Gao

**Affiliations:** 1 Department of Radiation Medicine, Faculty of Naval Medicine, Naval Medical University, 800 Xiangyin Road, Shanghai, China, 200433.; 2 Department of Radiology, Xizang Military General Hospital, 66 Niangre North Road, Lhasa City, Tibet, China, 850000.; 3 Department of Radiology, First Hospital of Jiaxing, Affiliated Hospital of Jiaxing University, 1882 Zhonghuan South Road, Jiaxing, Zhejiang, 314000.; 4 Department of Nuclear Radiation, Tongji University Affiliated Shanghai Pulmonary Hospital, 507 Zhengmin Road, Shanghai, China, 200433.

**Keywords:** Proanthocyanidins, lung cancer, radiosensitization, radioprotection, MAPK

## Abstract

Radiation-induced lung injury (RILI) is a common serious complication and dose-limiting factor caused by radiotherapy for lung cancer. This study was to investigate radioprotective effects of grape seed proanthocyanidins (GSP) on normal lung as well as radiosensitizing effects on lung cancer. *In vitro*, we demonstrated radioprotective effects of GSP on normal alveolar epithelial cells (MLE-12 and BEAS/2B) and radiosensitizing effects on lung cancer cells (LLC and A549). *In vivo*, we confirmed these two-way effects in tumor-bearing mice. The results showed that GSP inhibited tumor growth, and played a synergistic killing effect with radiotherapy on lung cancer. Meanwhile, GSP reduced radiation damage to normal lung tissues. The two-way effects related to the differential regulation of the MAPK signaling pathway by GSP on normal lung and lung cancer. Moreover, GSP regulated secretion of cytokines IL-6 and IFN-γ and expression of p53 and Ki67 on normal lung and lung cancer. Our findings suggest that GSP is expected to be an ideal radioprotective drug for lung cancer patients who are treated with radiotherapy.

## Introduction

Lung cancer ranks first in morbidity and mortality among malignant tumors 1, and radiotherapy is one of the main treatments for lung cancer. The incidence of radiation-induced lung injury (RILI) in patients with lung cancer after chest radiotherapy is 15% to 40% 2. The occurrence of RILI severely limits the dose of radiotherapy in the target area. Some patients even need to discontinue radiotherapy due to the occurrence and progressive exacerbation of RILI. Severe radiation-induced pulmonary fibrosis can even lead to death of patients 3, 4. Therefore, the protection of RILI caused by radiotherapy has extremely important medical significance. The main reason that some currently known radioprotective agents cannot be used in the clinic is that they prevent radiation damage to normal tissues and also have a radioprotective effect on tumor tissues, reducing the effect of radiotherapy 5-8.

Proanthocyanidin has obvious free radical scavenging ability, and its antioxidant activity in the body is 20 times that of VC and 50 times that of VE 9, 10. Free radicals play important roles in injuries induced by ionizing radiation. Our previous experiments have found that grape seed proanthocyanidins (GSP) have significant radioprotective effects on radiation pneumonitis and pulmonary fibrosis 11, 12. In addition, proanthocyanidins also have anti-tumor effects. The study of breast cancer 13, colorectal cancer 14, liver cancer 15, gastric cancer 16, oral squamous cell carcinoma 17, bladder cancer 18, skin cancer 19, kidney cancer 20, prostate cancer 21 and lung cancer 22 reported the obvious anti-tumor effects of proanthocyanidins. Therefore, when GSP is used for lung cancer patients who are treated with radiotherapy, it protects normal lung tissue against ionizing radiation and meanwhile it may enhance the killing effect of radiotherapy for lung cancer, which means that GSP is expected to be an ideal radioprotective drug for lung cancer patients who are treated with radiotherapy.

Based on different effects of proanthocyanidins on normal lung and lung cancer, this study conducted a preliminary discussion on its mechanism. Mitogen-activated protein kinase (MAPK) plays important roles in regulating proliferation, differentiation and apoptosis of cells 23. The MAPK/ERK signaling pathway is mainly related to cell proliferation and differentiation, while the MAPK/JNK and MAPK/p38 pathways are closely related to apoptosis of cells 23. Activation of MAPK/ERK signaling pathway promotes the growth of prostate cancer 24, cervical cancer 25, thyroid cancer 26, and breast cancer 27. MAPK/ERK-induced epithelial-mesenchymal transition promotes migration and metastasis of lung cancer cells 28. However, Blocking the MAPK/ ERK signaling pathway suppresses proliferation of human ovarian cancer cells 29 and prostate cancer cells 30. Unlike the MAPK/ERK pathway, activating the MAPK/JNK or MAPK/p38 pathway induces apoptosis of tumor cells, such as prostate cancer cells 24, breast cancer cells 31, 32, gastric cancer cells 33, and lung cancer cells 34. Anthocyanins induce apoptosis of colon cancer cells 35 and leukemic cells 36 by activating the MAPK/JNK or MAPK/p38 pathway. However, for normal cells, down-regulating the MAPK pathway protect them when they are physically or chemically damaged. Anthocyanins also protect normal cells by down-regulating the MAPK signaling pathway. Grape seed proanthocyanidins inhibit the activation of MAPK signaling pathway mediated by UV-induced oxidative stress in human epidermal keratinocytes 37. Grape seed proanthocyanidins played a similar effect in UV-exposed mouse skin when given to animals in the diet 38. Anthocyanins protect the retinal pigment epithelial cells from damage by suppressing the MAPK signaling pathway 39. Moreover, anthocyanins attenuate osteoarthritis 40, 41, encephalitis 42, 43, and skin inflammation 44 by inhibiting the MAPK signaling pathway. In this study, we investigated dual role of grape seed proanthocyanidins in normal lung and lung cancer after ionizing radiation, and found that the diverse regulation of MAPK signaling pathway account for the underlying mechanism.

## Materials and methods

### Cell culture and GSP treatment

All cells were from American Type Culture Collection. LLC (Mice Lewis lung cancer cells) and A549 cells (human non-small cell lung cancer cells) were cultured in DMEM medium and BEAS-2B (Human bronchial epithelial cells) and MLE-12 cells (mice lung epithelial cells) were cultured in RPMI 1640 medium. Both the DMEM and RPMI 1640 medium contained 10% fetal calf serum. Cell incubator kept at 37 °C with 5% CO2 and 95% humidity.

Grape seed proanthocyanidins (GSP) was obtained from Tianjin Peak Natural Products Research and Development co. LTD. (Tianjin, China). One hour before ionizing radiation, cells were pretreated with or without GSP-containing PBS. Twenty-four hours after treatment, cells were transferred to normal DMEM of RPMI 1640 medium.

### CCK-8 assay, colony formation assay and flow cytometric analysis

Cell viability was detected by CCK-8 assay (Cell Counting Kit-8; Dojindo, Kumamoto, Japan). Cell proliferation was detected by Colony formation assay as previous research 12. Cell apoptosis was detected by flow cytometric analysis using an Apoptosis Detection Kit (Invitrogen, Carlsbad, California, USA).

### Intracellular ROS measurement

The anti-oxidant (NAC) was used as a positive control to detect the scavenging effect of GSP on free radicals after irradiation. One hour before irradiation, GSP was given at a concentration of 20 ug/ml and NAC at a concentration of 10 mmol/L. Reactive Oxygen Species Fluorogenic Probe (Cat. No S0033; Beyotime; China) was used for measurement of intracellular ROS of A549 and BEAS-2B cells.

### Mice and GSP treatments

C57BL/6 6-week-old male mice were purchased from Shanghai Ling Chang biological technology co., LTD. All the experiments associated with mice were approved by the Laboratory Animal Center of Naval Medical University, Shanghai. Mice were used and randomly divided into six groups: two groups without lung cancer and other four groups with lung cancer. We injected 1×10^6^ Lewis lung carcinoma cells (LLC, ATCC) mixed with Matrigel (Matrigel: Medium, a ratio of 1:1, 25 μl total) into the left upper lobe of the mice to establish the lung cancer model. The lung cancer mice were divided into four groups, including a non-irradiated group, a GSP treatment group, an irradiation group and an irradiation with GSP treatment group. The lung cancer-free mice were divided into two groups, including a sham operation group and a simple Matrigel injected group. GSP (2 mg/ml) was delivered through drinking after LLC cells injected.

### Lung extraction and pathological staining

The left upper lobe lungs were resected and weighed at different times. The left upper lobe lungs, with or without cancers, were then used for pathological analysis and western blot analysis. HE staining and immunofluorescence staining was performed as previously described 45. Anti-P53 (1:500) and anti-Ki67 (1:500) antibodies were from Cell Signaling Tech, China.

After the upper lobe of the left lung of the mouse was embedded in paraffin, it was sectioned and stained every 0.5 mm with a microtome, and the largest cross section of the tumor was taken as the experimental result. The cross-sectional area of the tumor on the 4^th^, 7^th^, 11^th,^ 14^th^, 15^th^, 16^th^ and 18^th^ day after irradiation was compared with the cross-sectional area of the tumor on the 1st day after irradiation in each group, and the value obtained was used to compare the groups. In the same way, the weight changes of the upper left lung lobe between the groups were compared.

### ELISA assay

ELISA kits (Westang Tech., Shanghai, China) were used to detect the serum levels of IL-6 and IFN-γ.

### Western blot analysis

Proteins from cells and tissues were extracted by ProtecJETTM Mammalian Cell Lysis Reagent (Fermentas, Vilnius, Baltic, Lithuania). MAPK related antibodies (1:1000) were provided by Abcam Corporation. Other antibodies including the secondary antibody (1:1000) were provided by Cell Signaling Technology Corporation.

### Irradiation

LLC, A549, MLE-12 and BEAS-2B cells were exposed to^ 60^Co in the radiation center (Naval Medical University, Shanghai) with a dose of 8Gy at a dose rate of 1Gy/min. Local chest of all radiated mice were exposed to^ 60^Co with a dose of 25Gy at a dose rate of 1 Gy/min.

### Statistical analysis

Data were expressed as mean ± SD of three independent experiments and calculated using one-way ANOVA (Prism version 6.0 software). Student-Newman-Keuls post-hoc test was used to determine variance between groups. The difference between the groups was considered statistically significant when P < 0.05.

## Results

### Grape seed proanthocyanidins (GSP) sensitized lung cancer cells to ionizing radiation (IR) while alleviated radiation damage to normal lung cells

**Figure 1:**
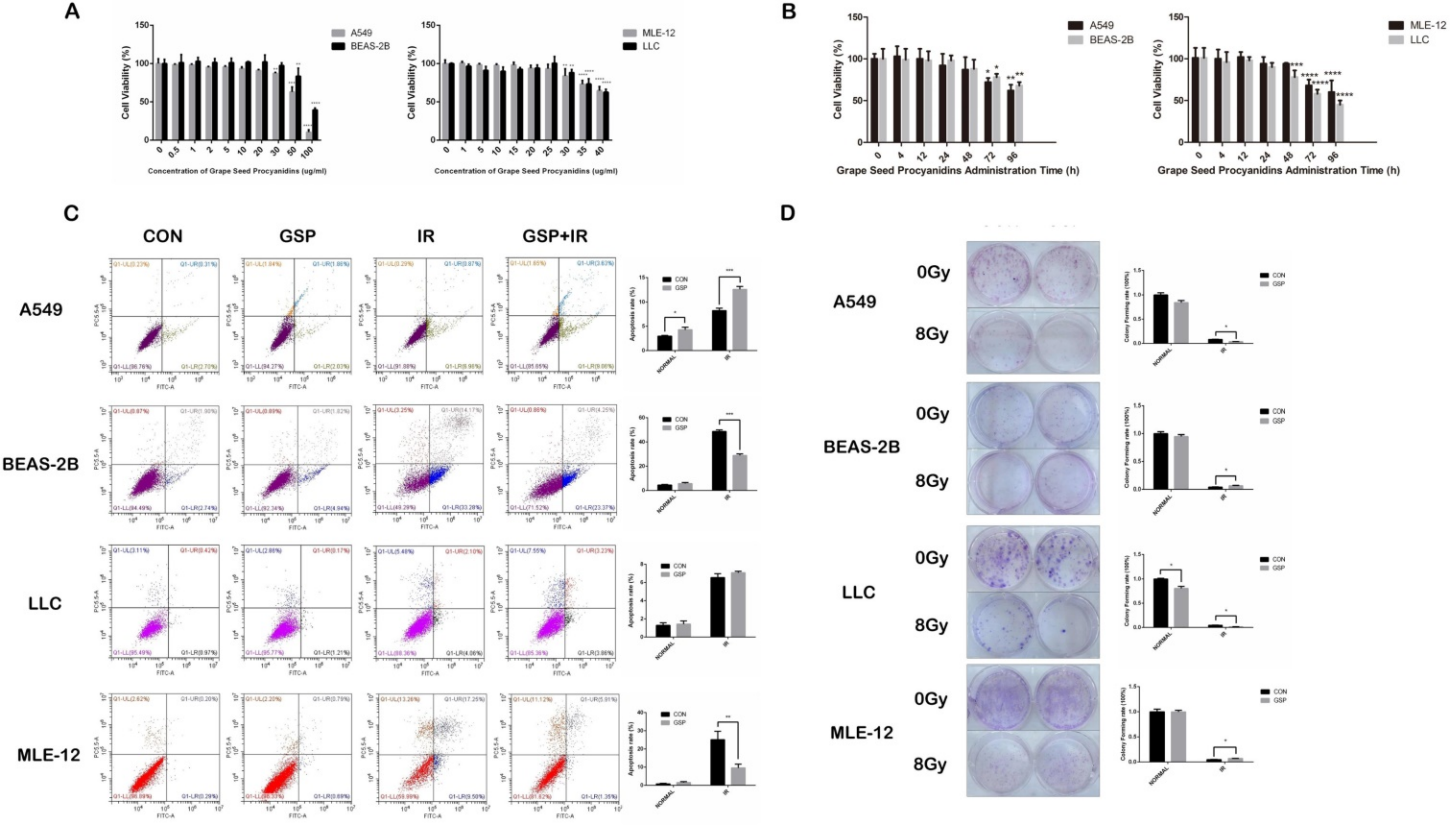
Grape seed proanthocyanidins (GSP) sensitizes lung cancer cells LLC and A549 to ionizing radiation (IR) while alleviates radiation damage to normal lung cells MLE-12 and BEAS-2B. (**A**) Cytotoxicity of different concentrations of GSP on lung cancer cells and normal lung cells. In CCK8 assay, no toxicity is found in both cancer cells (LLC and A549) and normal cells (MLE-12 and BEAS-2B) treated with GSP at the concentration less than 20 ug/ml. (**B**) Cytotoxicity of GSP on lung cancer cells and normal lung cells at different administration times. At the concentration of 20 ug/ml, no toxicity is found until 24 hours after administration. (**C**) In flow cytometry, compared with IR group, GSP treatment significantly decreases apoptosis in MLE-12 cells and BEAS-2B cells, while increases apoptosis in A549 cells. (**D**) The colony formation assay shows that GSP treatment significantly improves the viability of MLE-12 cells and BEAS-2B cells after irradiation, and reduces the viability of A549 after irradiation. LLC, A549, MLE-12 and BEAS-2B cells were exposed to ^60^Co with a dose of 8Gy at a dose rate of 1Gy/min. Data were presented as mean ± SD (n =3). *P<0.05, **P <0.01, ***P < 0.001, **** P < 0.0001.

In CCK8 assay, no toxicity was found in both cancer cells (LLC and A549) and normal cells (MLE-12 and BEAS-2B) treated with GSP at the concentration less than 20 ug/ml (Figure 1A). And at the concentration of 20 ug/ml, no toxicity was found until 24 hours after administration (Figure 1B). In flow cytometry, compared with IR group, GSP treatment significantly decreased apoptosis in MLE-12 cells and BEAS-2B cells, while increased apoptosis in A549 cells (Figure 1C). The colony formation assay confirmed that GSP treatment significantly improved the viability of MLE-12 cells and BEAS-2B cells after irradiation, and reduced the viability of A549 after irradiation (Figure 1D). Although no significant difference was found in LLC cells at a concentration of 20 ug/ml in flow cytometry analysis, clone formation assays showed that GSP reduced the viability of LLC cells after irradiation. In brief, we found that GSP sensitized lung cancer cells to IR while reducing radiation damage to normal lung cells.

### Suppressive and radiosensitive potential of GSP on tumor *in vivo*

**Figure 2:**
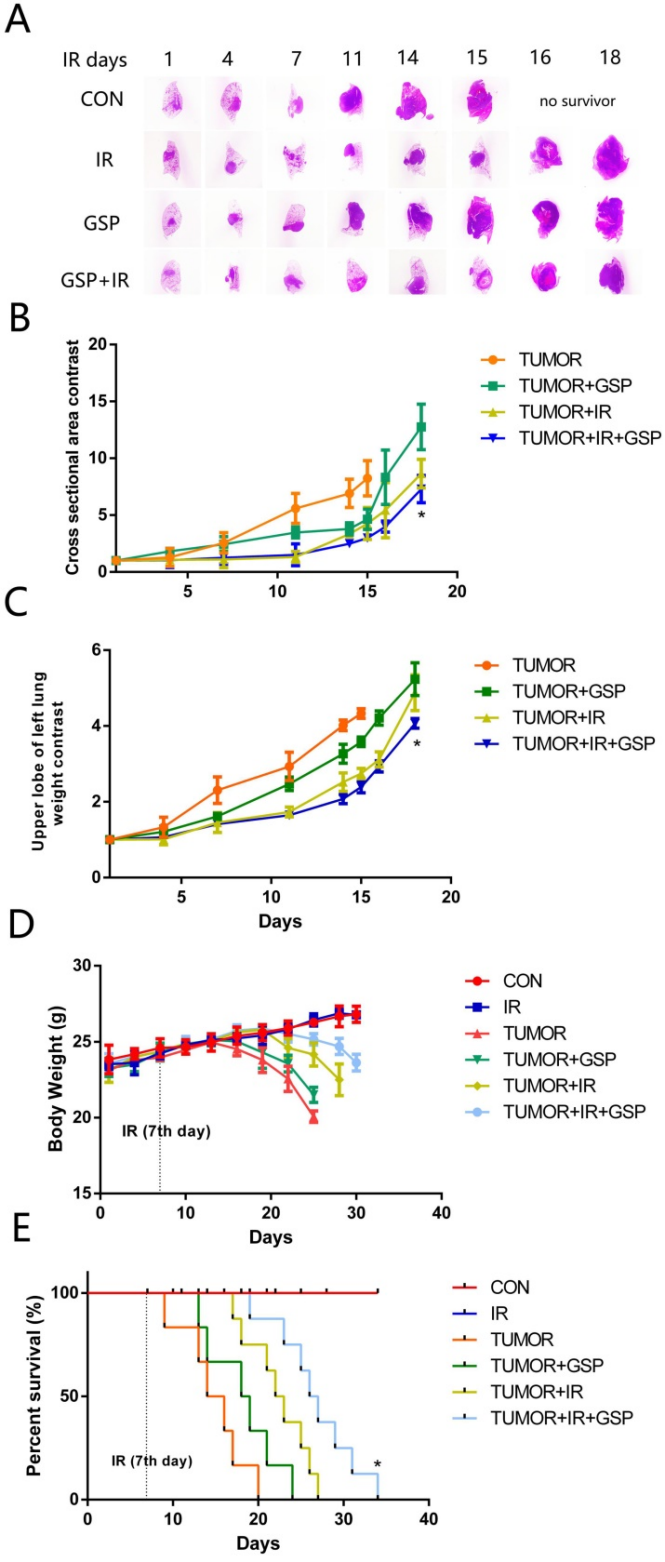
Suppressive and radiosensitive potential of GSP on tumor *in vivo*. (**A**) Representative images of HE stained sections of lung tissues with tumor. The largest cross section of the tumor is taken as the experimental result. IR, GSP, and IR+GSP reduce tumor size in tumor-bearing mice. (**B**) The cross-sectional area of the tumor on the 4^th^, 7^th^, 11^th,^ 14^th^, 15^th^, 16^th^ and 18^th^ day after irradiation is compared with the cross-sectional area of the tumor on the 1st day after irradiation in each group, and the value obtained is used to compare the groups. The results show that IR + GSP has the most obvious inhibitory effect on tumor. (**C**) In the same way, the weight changes of the upper left lung lobe between the groups are compared. IR, GSP, and IR+GSP reduce the weight of the lung lobe where the tumor is located. (**D**) IR, GSP, and IR+GSP improve the adverse effect of lung cancer on body weight, most significant in IR+GSP group. (**E**) IR, GSP, and IR+GSP improve the survival rate of tumor-bearing mice, most significant in IR+GSP group. Local chest of all radiated mice were exposed to ^60^Co with a dose of 25Gy at a dose rate of 1 Gy/min. GSP (2 mg/ml) was delivered through drinking after LLC cells injected. Data were presented as mean ± SD (n=3). *P<0.05, **P <0.01, ***P < 0.001, **** P < 0.0001.

To mimic the microenvironment of lung cancer, we injected LLC cells into the left upper lobe of C57BL/6 mice. We found that IR, GSP, and IR+GSP reduced tumor volume in tumor-bearing mice (Figure 2A, 2B), reduced the weight of the lung lobe where the tumor was located (Figure 2C), improved the adverse effect of lung cancer on body weight (Figure 2D) and increased survival (Figure 2E). The effect of IR+GSP was stronger than IR and GSP, indicating that GSP had the effect of inhibiting tumor growth, and played a synergistic effect with IR to kill tumor cells.

### GSP attenuated inflammation of normal lung tissue and promoted apoptosis of tumor after irradiation

**Figure 3:**
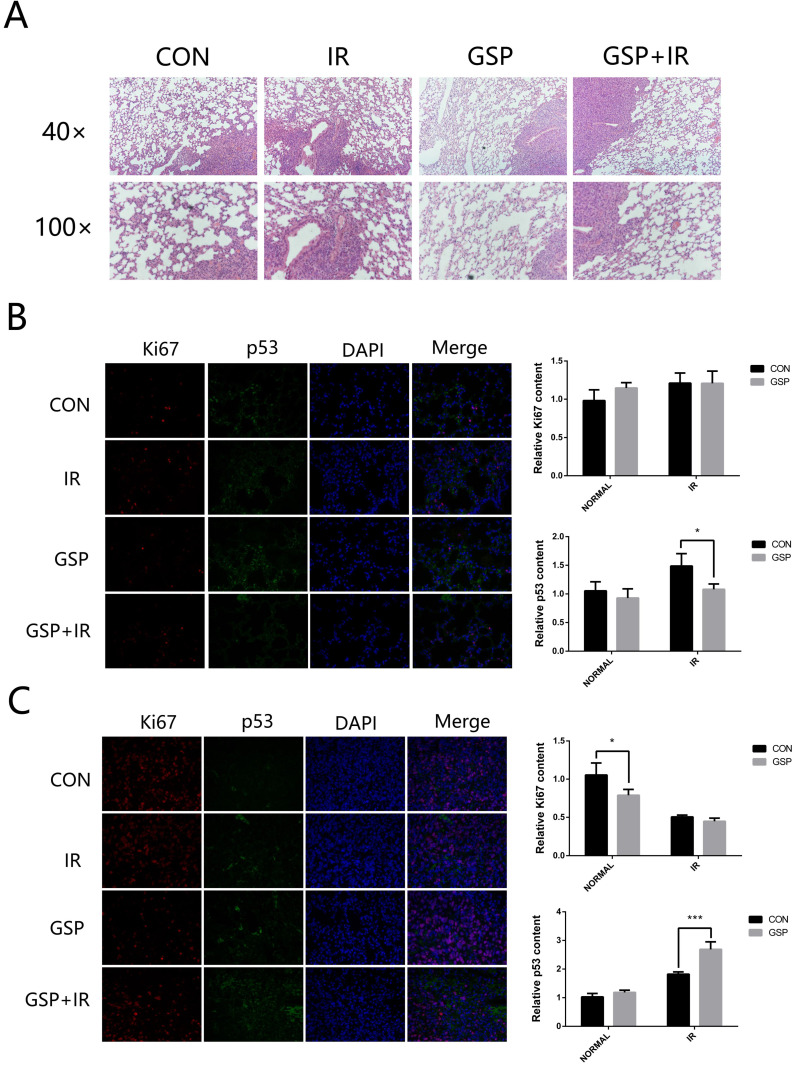
GSP attenuates inflammation of normal lung tissues and promotes apoptosis of tumor after irradiation. (**A**) Representative images of HE stained sections of lung tissues with tumor. GSP treatment markedly attenuates the inflammation in normal lung tissue surrounding the tumor. After local chest ionizing radiation, a large number of inflammatory cells accumulate in normal lung tissue, and the extracellular matrix is excessively deposited. The normal structure of the alveoli is destroyed, with thickened alveolar wall. However, GSP treatment significantly alleviates these changes. (**B**) Representative images and a quantification of Ki67 and P53 expressions in normal lung tissues by immunohistochemical staining. Ionizing radiation increases the expression of P53 in normal lung tissues, while GSP reduces the increment of P53 expression. GSP has no effect on the expression of Ki67 in normal lung tissues. (**C**) Representative images and a quantification of Ki67 and P53 expressions in tumor by immunohistochemical staining. Ionizing radiation increases the expression of P53 in lung cancer tissues, and GSP further increases the expression of P53. In addition, GSP down-regulates the expression of Ki67 in lung cancer tissues. Data were presented as mean ± SD (n=3). *P<0.05, **P<0.01, ***P<0.001, **** P < 0.0001.

From the HE stained sections, inflammation was found in normal lung tissue surrounding the tumor (Figure 3A). GSP treatment markedly attenuated the inflammation. After local chest ionizing radiation, a large number of inflammatory cells accumulated in normal lung tissue, and the extracellular matrix was excessively deposited. The normal structure of the alveoli was destroyed, with thickened alveolar wall. However, GSP treatment significantly alleviated these changes.

Immunohistochemical staining showed that ionizing radiation increased the expression of P53 in both normal lung tissues and lung cancer tissues, but GSP reduced the increment of P53 expression in normal lung tissues (Figure 3B) while further increase the expression of P53 in lung cancer tissues (Figure 3C). In addition, we found that GSP down-regulated the expression of Ki67 in lung cancer tissues while it had no effect on normal lung tissues.

### GSP regulated secretion of cytokines IL-6 and IFN-γ after ionizing radiation

**Figure 4:**
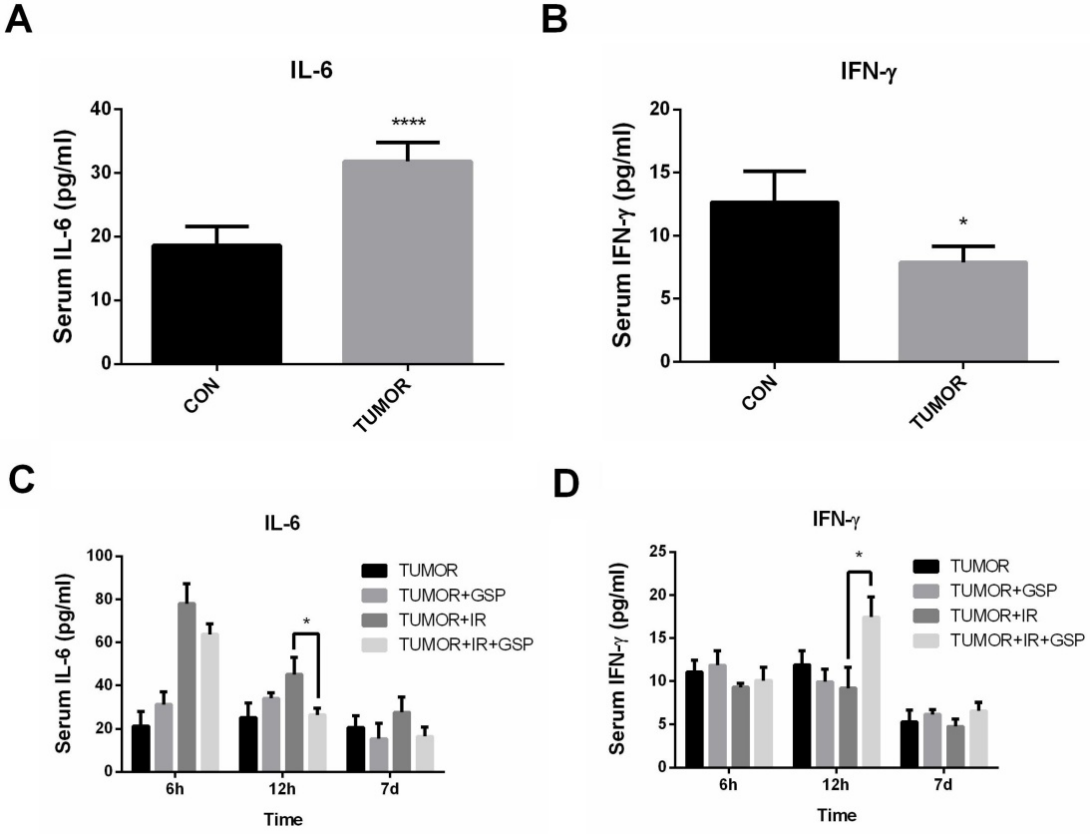
GSP regulates secretion of cytokines IL-6 and IFN-γ after ionizing radiation. Compared with the normal mice, tumor-bearing mice have significantly higher IL-6 levels (**A**) and lower IFN-γ levels (**B**). At 12 hours after irradiation, the serum IL-6 level in the IR+GSP mice is significantly lower than that of IR mice (**C**), while IFN-γ levels are significantly increased (**D**). Data were presented as mean ± SD (n=3). *P<0.05, **P <0.01, ***P < 0.001, **** P < 0.0001.

Tumor-bearing mice had significantly higher IL-6 levels and lower IFN-γ levels compared with the normal mice (Figure 4A, 4B). The serum of tumor-bearing mice after local chest irradiation was detected, and the serum IL-6 level was significantly increased 6 hours after irradiation. At 12 hours after irradiation, the serum IL-6 level in IR+GSP mice was significantly lower than that of IR mice, while IFN-γ levels were significantly increased (Figure 4C, 4D).

### GSP reduced ROS levels in both cancer cells A549 and normal cells BEAS-2B

**Figure 5:**
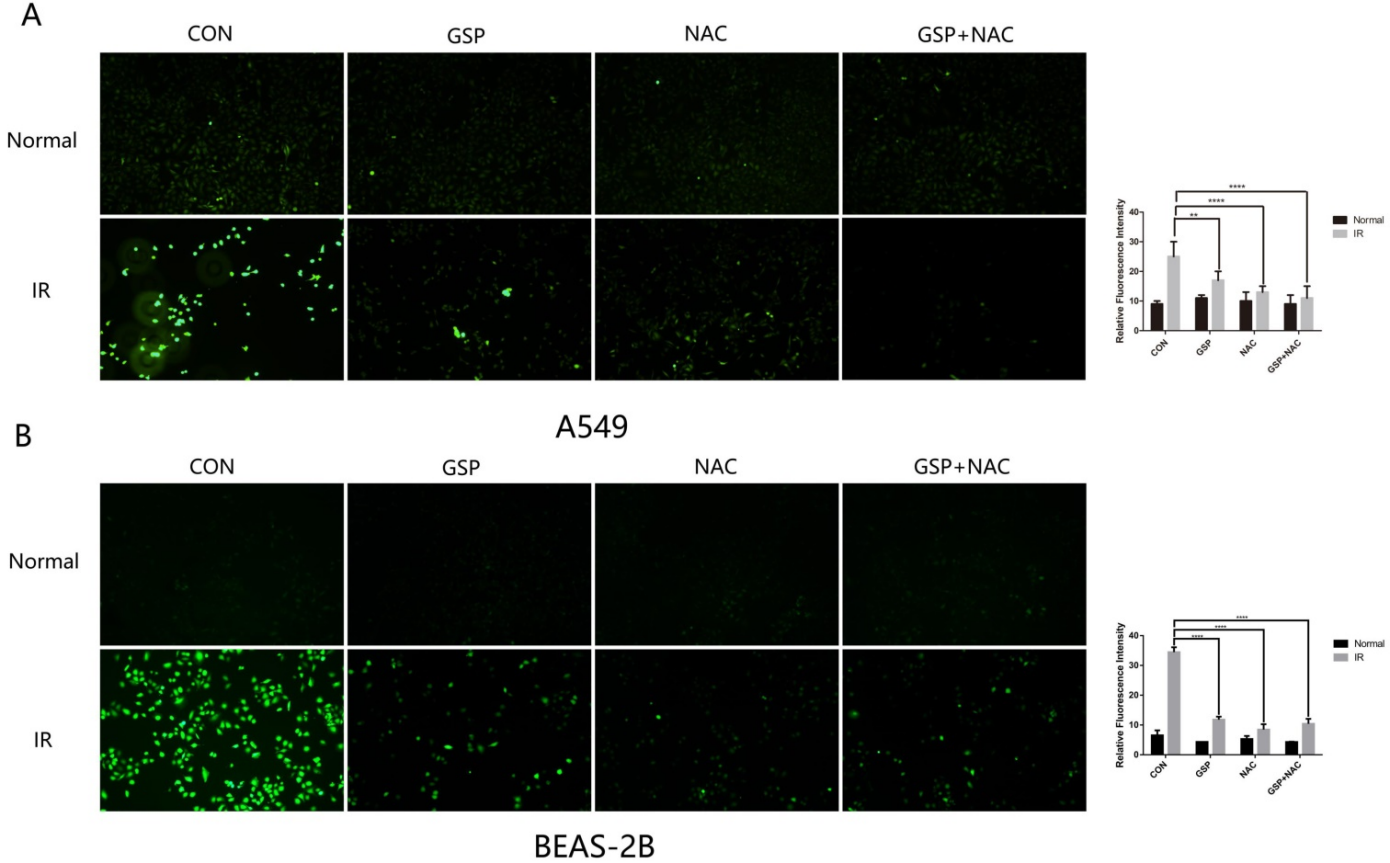
GSP reduces ROS levels in both cancer cells A549 and normal cells BEAS-2B. The anti-oxidant (NAC) was used as a positive control to detect the scavenging effect of GSP on free radicals after irradiation. One hour before irradiation (8Gy, 1Gy/min), GSP were given at a concentration of 20 ug/ml and NAC at a concentration of 10 mmol/L. The intracellular ROS level of A549 and BEAS-2B cells were detected after irradiation by Reactive Oxygen Species Fluorogenic Probe and were observed with confocal microscopy. GSP treatment significantly reduces ROS levels in both cancer cells A549 (**A**) and normal cells BEAS-2B (**B**). Data were presented as mean ± SD (n=3). *P<0.05, **P <0.01, ***P < 0.001, **** P < 0.0001.

The anti-oxidant (NAC) was used as a positive control to detect the scavenging effect of GSP on free radicals after irradiation. The intracellular ROS level of A549 and BEAS-2B cells were detected after irradiation by Reactive Oxygen Species Fluorogenic Probe. We found that GSP treatment significantly reduced ROS levels in both cancer cells A549 (Figure 5A) and normal cells BEAS-2B (Figure 5B).

### GSP differentially regulated MAPK signaling pathways in lung cancer cells/tissues and normal lung cells/tissues

**Figure 6:**
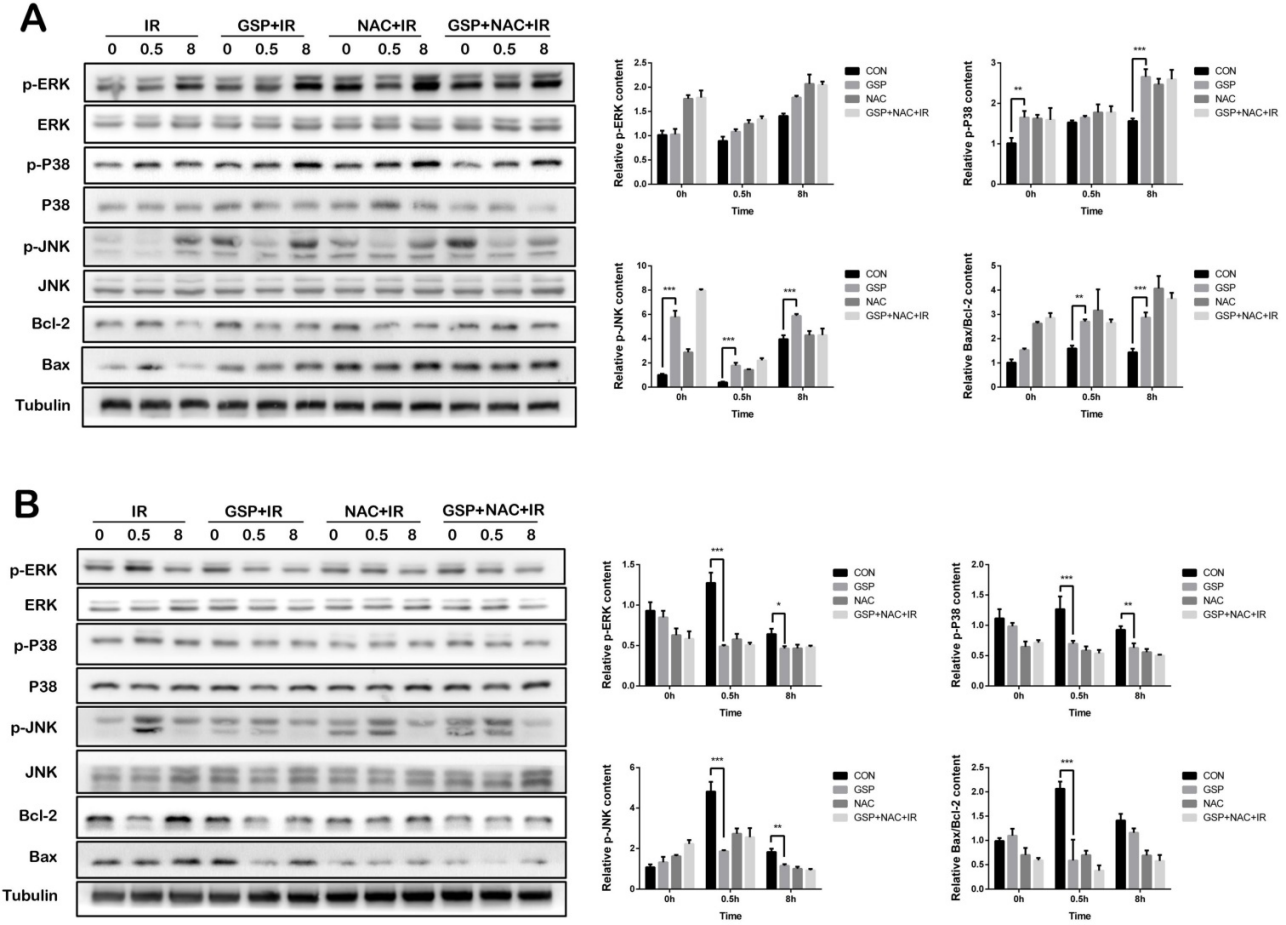
GSP differentially regulates MAPK signaling pathways in lung cancer cells and normal lung cells respectively. (**A**) In lung cancer cells A549, GSP significantly increases the expression of p-JNK, p-P38 protein before and after irradiation, and the value of Bax/Bcl-2 after irradiation, and has less effect on p-ERK protein. (**B**) In normal lung epithelial cells BEAS-2B, GSP reduces the expression of p-JNK, p-P38, and p-ERK proteins of MAPK family after irradiation, and reduces the expression of Bax and value of Bax/ Bcl-2. The raw density of Western blot figures was quantified. Data were presented as mean ± SD (n=3). *P<0.05. **P <0.01, ***P < 0.001, **** P < 0.0001.

In lung cancer cells A549, GSP significantly increased the expression of p-JNK, p-P38 protein before and after ionizing radiation, and the value of Bax/Bcl-2 after irradiation, and had less effect on p-ERK protein (Figure 6A). In normal lung epithelial cells BEAS-2B, GSP reduced the expression of p-JNK, p-P38, and p-ERK proteins of MAPK family after ionizing radiation, and reduced the expression of Bax and value of Bax/ Bcl-2 (Figure 6B).

**Figure 7:**
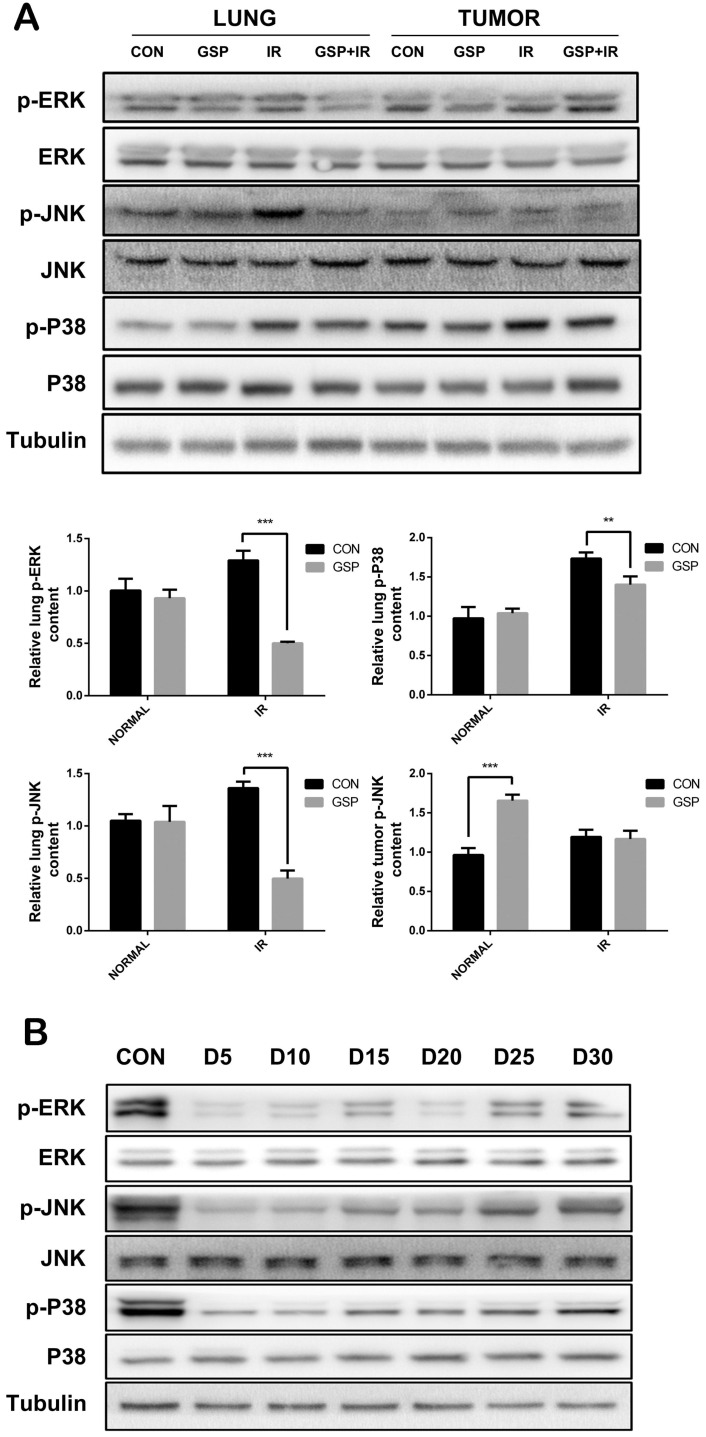
GSP differentially regulates MAPK signaling pathways in lung cancer tissues and normal lung tissues. (A) In tumor-bearing mice, ionizing radiation increases expression of p-JNK, p-P38, and p-ERK proteins of MAPK family in normal lung tissues, while GSP reduces the increments. In lung cancer tissues, GSP activates the expression of p-JNK protein. (B) In normal mice, the expression of p-JNK, p-P38, and p-ERK proteins of MAPK family in the lung tissues is significantly reduced when GSP is given in diet, but the protein expression gradually increases over the feeding time. The raw density of Western blot figures was quantified. Data were presented as mean ± SD (n=3). *P<0.05. **P <0.01, ***P < 0.001, **** P < 0.0001.

In tumor-bearing mice, ionizing radiation increased expression of p-JNK, p-P38, and p-ERK proteins of MAPK family in normal lung tissues, while GSP reduced the increments. In lung cancer tissues, GSP activated the expression of p-JNK protein (Figure 7A). In normal mice, the expression of p-JNK, p-P38, and p-ERK proteins of MAPK family in the lung tissue was significantly reduced when GSP was given in diet, but the protein expression gradually increased over the feeding time (Figure 7B).

## Discussion

Radiotherapy is one of the main treatments for lung cancer. Radiation-induced lung injuries (RILI) are common serious complications and dose limiting factors of radiotherapy for lung cancer. One of the main reasons for limiting the clinical application of radioprotective drugs for lung cancer patients who are treated with radiotherapy is that existing radioprotective drugs have protective effects on both normal lung tissue and lung cancer tissue. In this study, a model of lung cancer-bearing mice was established, and the results showed that grape seed proanthocyanidins (GSP) had a radioprotective effect on normal lung tissue and a radiosensitizing effect on lung cancer tissue. Therefore, GSP is expected to be a very ideal radioprotective drug for lung cancer patients who are treated with radiotherapy.

Proanthocyanidins have obvious antioxidative ability, and have good protective effect on oxidative stress related diseases. Proanthocyanidins have protective effects on UV-B-induced oxidative damage to keratinocytes through activation of Nfr2 signaling 46. Proanthocyanidins protect renal tubular cell against apoptosis induced by oxidative stress in mice 47. Proanthocyanidins significantly increase the content of superoxide dismutase and peroxidase in liver tissues after ionizing radiation, thereby significantly protecting the liver tissues of exposed rats 48. Other researchers reported that proanthocyanidins increase the number of peripheral blood lymphocytes in 6Gy-irradiated rats and reduce the damage to the antioxidant system caused by free radicals 49. Our previous experiments demonstrated that proanthocyanidins have radioprotective effects on RILI in normal mice 12. This study confirmed the protective effect of proanthocyanidins on RILI in a lung cancer-bearing mouse model. In addition, proanthocyanidins also played a synergistic killing effect on lung cancer tissues, improving the radiotherapy effect. Compared with the IR and GSP groups, the IR + GSP group had better cancer suppression effect and survival rate. The results of HE and immunofluorescence staining showed that GSP reduced the inflammatory response of normal lung tissues, while enhancing the killing effect of radiotherapy on lung cancer. The antitumor effects of proanthocyanidins have been reported. The study of breast cancer 13, colorectal cancer 14, liver cancer 15, gastric cancer 16, oral squamous cell carcinoma 17, bladder cancer 18, skin cancer 19, kidney cancer 20, prostate cancer 21 and lung cancer 22 reported the obvious antitumor effects of proanthocyanidins. This study revealed that proanthocyanidins protected normal lung tissue against ionizing radiation and sensitized lung cancer tissues to ionizing radiation simultaneously. We observed similar results in experiments *in vitro*. Through flow cytometry and colony formation analysis, we found that proanthocyanidins had radiosensitizing effects on lung cancer cells LLC and A549, but had radioprotective effect on normal cells MLE-12 and BEAS-2B.

Proanthocyanidins also have significant anti-inflammatory effects. Proanthocyanidins can improve encephalitis 42, 43, Osteoarthritis 40, 41 and skin inflammation 44. The imbalance of Th1/Th2 was an important feature for the inflammatory response of the body. IL-6 is mainly secreted by Th2 cells or macrophages. IL-6 involved in RILI as a pro-inflammatory cytokine, and elevated serum IL-6 levels in patients can be used as independent predictors of clinical RILI 50. In addition, increased serum IL-6 expression stimulates the growth of cancer cells 51. IFN-γ is mainly secreted by Th1 cells and induces cancer cells apoptosis by activating caspase-8 and JNK-STAT 52. IFN-γ inhibits the metastasis and metabolism of cancer cells by reducing VEGF, TGF-α and other pro-angiogenic factors 53. Our previous research results showed that GSP regulated the secretion of Th1/Th2-related cytokines in normal mice 12. In this study, the GSP + IR group significantly inhibited IL-6 levels and increased IFN-γ levels 12 hours after irradiation in lung cancer-bearing mice, suggesting that GSP played a role in mitigating inflammation and suppressing lung cancer.

The presence of reactive oxygen free radicals (ROS) caused by ionizing radiation was one of the important mechanisms causing the damage of cells and tissues. GSP, as a substance rich in phenolic hydroxyl, could rapidly neutralize a large amount of free radical oxygen ions and reduce radiation damage. In this study, we found that GSP significantly reduced ROS levels in normal cells BEAS-2B after irradiation. In cancer cells A549, GSP also reduced intracellular ROS levels, which may protect lung cancer cells against ionizing radiation to a certain extent. However, what we had observed was that GSP had a synergistic killing effect on tumor cells with radiotherapy. Therefore, the differential effect of GSP on normal cells and cancer cells does not work through its antioxidant capacity.

The mitogen-activated protein kinase (MAPK) is a specific type of silk/serine kinase, and composed of five parallel signaling pathways. The biological effects of each signal pathway are different due to their different distribution and activation degree in different tumor cells. Ionizing radiation results in an increase in ROS, activating the expression of MAPK pathway 54, and increases the expression of inflammatory cytokines 55. In addition, this study found that irradiation activated the MAPK signaling pathway. However, in normal cells BEAS-2B, GSP significantly inhibited the expression of p-JNK, p-ERK and p-P38, thereby protecting BEAS-2B cells against ionizing radiation. Proanthocyanidins have been reported to protect normal cells via down-regulating the MAPK signaling pathway. The study by Lee MH 44 has shown that proanthocyanidins-rich rose petal extract protects normal skin via down-regulating the MAPK signaling pathway. Proanthocyanidins also protect retinal pigment epithelial cells from UVB-induced damage by down-regulating the MAPK signaling pathway 39. However, in human lung cancer cells A549, GSP significantly enhanced the phosphorylation of JNK protein after irradiation, thereby enhancing the killing effect of radiotherapy on A549 cells in this study. JNK activation promotes apoptosis 56, 57 and autophagy 58 of cancer cells. Alnus hirsuta extract exerts anti-cancer activity against MCF-7 cells *in vitro* by activating JNK 31. Bronchine D induces apoptosis and autophagy of lung cancer cell by up-regulating JNK phosphorylation levels *in vitro* and *in vivo* 34. In contrast, down-regulating JNK phosphorylation promotes cancer cells survive 24.

## Conclusion

This study showed that grape seed proanthocyanidins (GSP) had a radioprotective effect on normal lung tissue and a synergistic killing effect with radiotherapy on lung cancer tissues, which related to the differential regulation of the MAPK signaling pathway by GSP on normal lung and lung cancer. Moreover, GSP regulated secretion of cytokines IL-6 and IFN-γ and expression of p53 and Ki67 on normal lung and lung cancer. Our findings suggest that GSP is expected to be an ideal radioprotective drug for lung cancer patients who are treated with radiotherapy.
